# Biofilm growth and microbial contamination of dental unit waterlines at Kuwait University dental center

**DOI:** 10.3389/froh.2022.1071018

**Published:** 2023-01-09

**Authors:** Jaber Hussain Akbar, Jawad Behbehani, Maribasappa Karched

**Affiliations:** ^1^ Department of Restorative Sciences, Faculty of Dentistry, Kuwait University, Kuwait City, Kuwait; ^2^ Department of Bioclinical Sciences, Faculty of Dentistry, Kuwait University, Kuwait City, Kuwait

**Keywords:** biofilm, microbial contamination, dental unit water line, pathogenic species, 16S rRNA gene sequencing

## Abstract

Biofilm formation in dental unit waterlines and the resulting microbial contamination of the water in the system has become a significant problem. Contaminated water in the dental units is a major concern in dental clinics due to potential risk of causing infections particularly in elderly and immunocompromised patients. The aim of this study was at first to determine microbial contamination of the dental unit waterlines and then to study the efficacy of a comprehensive disinfection protocol on decreasing the microbial load. Water samples were collected before and after disinfection procedure from handpieces and water storage bottles from the dental units, a small 1-cm tubing was cut from each unit and subjected to microbiological culture on different growth media. Identification of the predominant species was achieved by 16S rRNA gene sequencing. Microbial growth was observed in samples collected from all dental units. Upon disinfection procedure, microbial contamination in the water samples and in the tubing surfaces was significantly reduced (*P* > 0.05). 16S rRNA gene sequencing revealed the presence of several species belonging to the genera *Staphylococcus*, *Corynebacterium* and *Roseomonas*, some of which are implicated in human infections. Aggravation of the biofilm growth on the tubing surfaces and the microbial contamination in the water can be effectively controlled by implementing appropriate and routine disinfection protocols. This may help protect the dental unit staff and the patients being exposed to the risk of infections.

## Background

Biofilm formation inside or on the surface of medical devices is a serious public health issue. It has been shown by several studies that Dental Unit Water Line (DUWL) harbor bacterial biofilms (1–3). Dental chair associated tools such as 3-in-1 syringe, air rotors, scalers etc. may receive heavy loads of microorganisms, thus providing a potential source of infection putting both practice staff and patients at risk. Microorganisms grow as multispecies biofilm on inner surfaces of water tubing. A fundamental property of biofilms is that they resist penetration by antimicrobial agents and the bacteria inside the biofilms are more resistant to antimicrobials than the planktonic cells (4, 5). Despite regular use of disinfectants in DUWL, biofilms have been reported to persist and proliferate (6). Therefore, a comprehensive microbiological analysis of water and biofilm in DUWL is essential in managing microbial contamination of DUWL.

The ability of microorganisms to attach to surfaces is a critical factor in biofilm initiation and growth. Microorganisms grow and persist inside the DUWL on the inner surface of the tubing. Once a mature biofilm is formed, it is extremely difficult to dislodge it since biofilm is resistant to antimicrobials. Planktonic bacteria are continually released from these mature biofilms into the water flow in DUWL. Microorganisms colonizing DUWL are generally non-pathogenic aerobic and heterotrophic environmental bacteria. However, occasionally, pathogenic and/or opportunistic pathogens such as *Pseudomonas*, *Legionella* and *Mycobacterium* species are found (7, 8).

Manufacturers of dental units generally provide recommendations to keep the DUWL contamination-free. Little is known in the literature about the effectiveness of those recommendations. Furthermore, there are no evidence-based standard guidelines to prevent DUWL contamination. The aim of this study was to examine microbial biofilm formation on the inner surface of DUWL tubing, microbial contamination of the waterlines, and subsequently to assess the efficacy of a comprehensive disinfection protocol on controlling the microbial growth in the DUWL.

## Methods

Methods for sampling of DUWL and subsequent microbiological analyses were adapted from previously published literature (9, 10).

### Sampling of DUWL

A total of 12 dental units, 6 from A-dec, and 6 from KaVo were investigated. Two water and one biofilm sample were collected from each unit before and after treatment with disinfectant: (1) waterline sample from a high-speed hand piece that delivers water into the patients' mouth during treatment, (2) sample from the source water supplied to the DUWL, and 3) for biofilm analysis, a 5-cm piece of DUWL tubing.

Water samples were processed using the following method: One hundred ml of water samples “(1)” and “(2)” above were collected in sterile flasks containing 0.1 g sodium thiosulfate (to inactivate any residual disinfectant) and transported to Oral Microbiology Laboratory immediately on ice. Samples were filtered through Sterifil® Aseptic System, a 0.2 µm sterile vacuum filter (Millipore). The membrane was then removed using sterile forceps and placed in a sterile screw cap tube containing 10 ml sterile phosphate buffered saline (PBS). Organisms bound to membrane were collected into PBS by vigorously vortexing at maximum speed for 1–2 min.

Biofilm samples were collected as follows: External surfaces of the DUWL tubing were wiped with 70% ethanol and approximately 5 cm of the tubing was cut using sterile scissors. The tubing sections were placed in sterile screw cap tubes containing a small volume of sterile water to cover the specimen. The samples were immediately transported to the lab on ice. The tubing was sectioned vertically under aseptic conditions to expose inner surface. Planktonic non-adherent bacteria were removed by rinsing the tubing sections in PBS. Using sterile dental probes, biofilm formed on the surface of the specimen was collected in 1 ml sterile PBS in microfuge tubes.

### Total viable counts (TVC) in water and biofilm samples

Water and biofilm samples were processed for microbiological culture within 30 min of collection. Serial 10-fold dilutions (up to 10^−5^) of the samples were prepared in sterile PBS and plated on various selective and non-selective culture media. The following media were used: (1) Brucella blood agar for *Actinomyces* spp., Streptococci and other oral anaerobes. Incubation anaerobically (80% nitrogen, 10% CO_2_ and 10% hydrogen) at 37 °C for 7–10 days. (2) R2A agar for environmental bacteria (11). Aerobic incubation at 37 °C for 2–3 days. (3) Pseudomonas agar with CFC supplement. Aerobic incubation at 37 °C for 2 days. (4) MacConkey II Agar for enterobacteria. Aerobic incubation at 37 °C for 2 days. (4) Sabouraud Dextrose Agar (SDA) for Candida spp. Aerobic incubation at 37 °C for 2 days.

### Culture of control bacterial strains

To ensure that various culture media and the incubation conditions were appropriate, *Escherichia coli* EC49, *Pseudomonas aeruginosa* PS52, *Streptococcus mutans* NCTC 10449, *Actinomyces naeslundii* ATCC 12104, *Candida albicans* AE-112 were cultured on respective growth media each time when dental unit samples were cultured.

### Treatment regimens

The American Dental Association recommends that the bacterial counts in dental unit waterlines should not exceed 200 CFU/ml (12). As the counts were higher than this at KUDC dental units we implemented a comprehensive treatment of the waterlines based on recommendations in published literature with some modifications (13). Water in the dental unit water storage bottle was completely removed and treated with Oxygenal for 10 min. The dental unit tubings and syringe were also flushed with Oxygenal as per the user instructions. Fresh distilled water was supplied daily, and the left-over water was discarded each day. All the water that was left in the waterlines was drained off at the end of the day. Hand piece, three-in-one syringe and other instruments attached to the dental unit waterline were flushed for 5 min at the beginning of each day to remove any bacteria remaining in the waterline tubing. “After-treatment” samples were collected after 2 weeks.

### Scanning electron microscopy

Approximately 1-cm length of tubing that were already cut vertically into two halves were processed for scanning electron microscopy. The pieces were at first fixed in 3% glutaraldehyde for 3 1/2 h at room temperature and then transferred to buffer and kept at 4 °C until further processing was done. The tubing samples were removed from the buffer, washed thoroughly with purified water. The tubing samples were then mounted on sample holders. The samples were then dried completely in a critical point dryer, mounted on stubs with carbon double adhesive tape, gold-coated, and stored in a desiccator until observation. The samples were examined on the scanning electron microscope JSM IT 200 (JEOL, Japan).

### DNA purification

Genomic DNA from the bacterial colonies was purified by using DNeasy Blood and Tissue kit (Qiagen Inc., Valencia, CA) following the manufacturer's instructions. Briefly, bacterial suspensions were treated with lysis buffer and proteinase-K at 56 °C for 1 h. DNA from lysed samples were applied onto a spin column, washed and eluted with elution buffer or sterile water for all samples. This DNA was used as template in PCR amplification.

### Identification of species by 16s rRNA gene sequencing

From microbial cultures, two to three colonies representing each colony type on all growth media were selected for identification by colony PCR of complete 16S rRNA gene followed by sequencing of the V4 region as described (14). Complete 16S rDNA sequence of ∼1500 bp was amplified using universal forward primer D88 (5′-GAGAGTTTGATYMTGGCTCAG) and reverse primer E94 (5′-GAAGGAGGTGWTCCARCCGCA). Ready-To-Go® PCR beads were used for PCR amplification. After an initial denaturation at 95 °C for 10 min, 30 cycles of 95 °C for 1 min, 50 °C for 30 s and 72 °C for 30 s were run followed by a final elongation of 72 °C for 5 min. A small aliquot of each PCR product was first verified on a 1% agarose gel and the remainder purified using QiaQuick® PCR purification spin columns (Qiagen). Purified amplicons were sequenced by using primersF17 and B34 and a BigDye Terminator ® kit on Beckman Coulter CEQ8000 sequencer. Sequence data obtained was used for similarity search at NCBI BLAST.

### Statistical analyses

Bacterial quantities were log transformed after adding 1 to all data to handle zeroes. Normality of the data was tested by Skewness and Kurtosis values, Shapiro Wilkins *P* values and histograms. Nonparametric Mann Whitney U test was used to compare the groups. A *P* value <0.05 was considered significant.

## Results

### Bacterial contamination of DUWL

**Figure 1:**
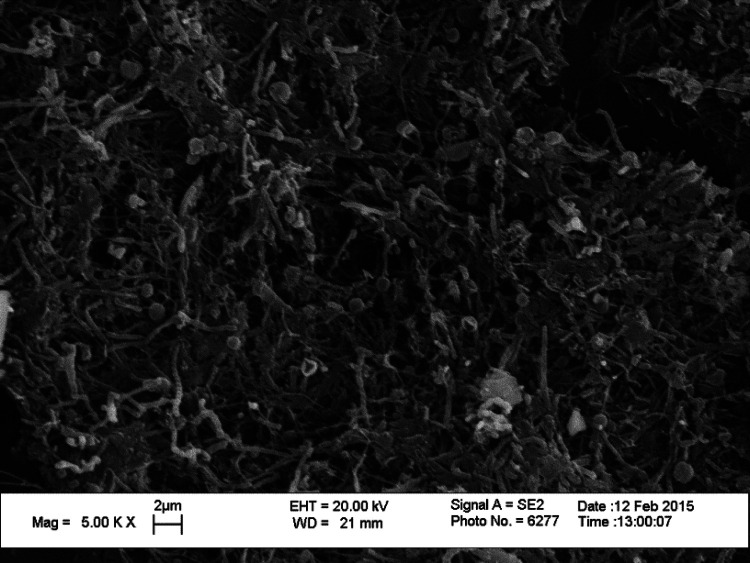
Scanning electron micrograph of the inner surface of DUWL tubing. A thick biofilm mass is seen on the inner surface of the tubing.

Bacterial contamination was assessed in two different types of DUWL, A-dec and KaVo. Microbiological analysis of water samples from the handpieces and water storage bottles was performed before and after decontamination procedures. As evident from the scanning electron microscopy image in Figure 1, a dense biofilm was observed on the luminal surface of the DUWL tubing, revealing a variety of rods and cocci in the biofilm matrix.

### Waterline contamination of handpieces

**Figure 2:**
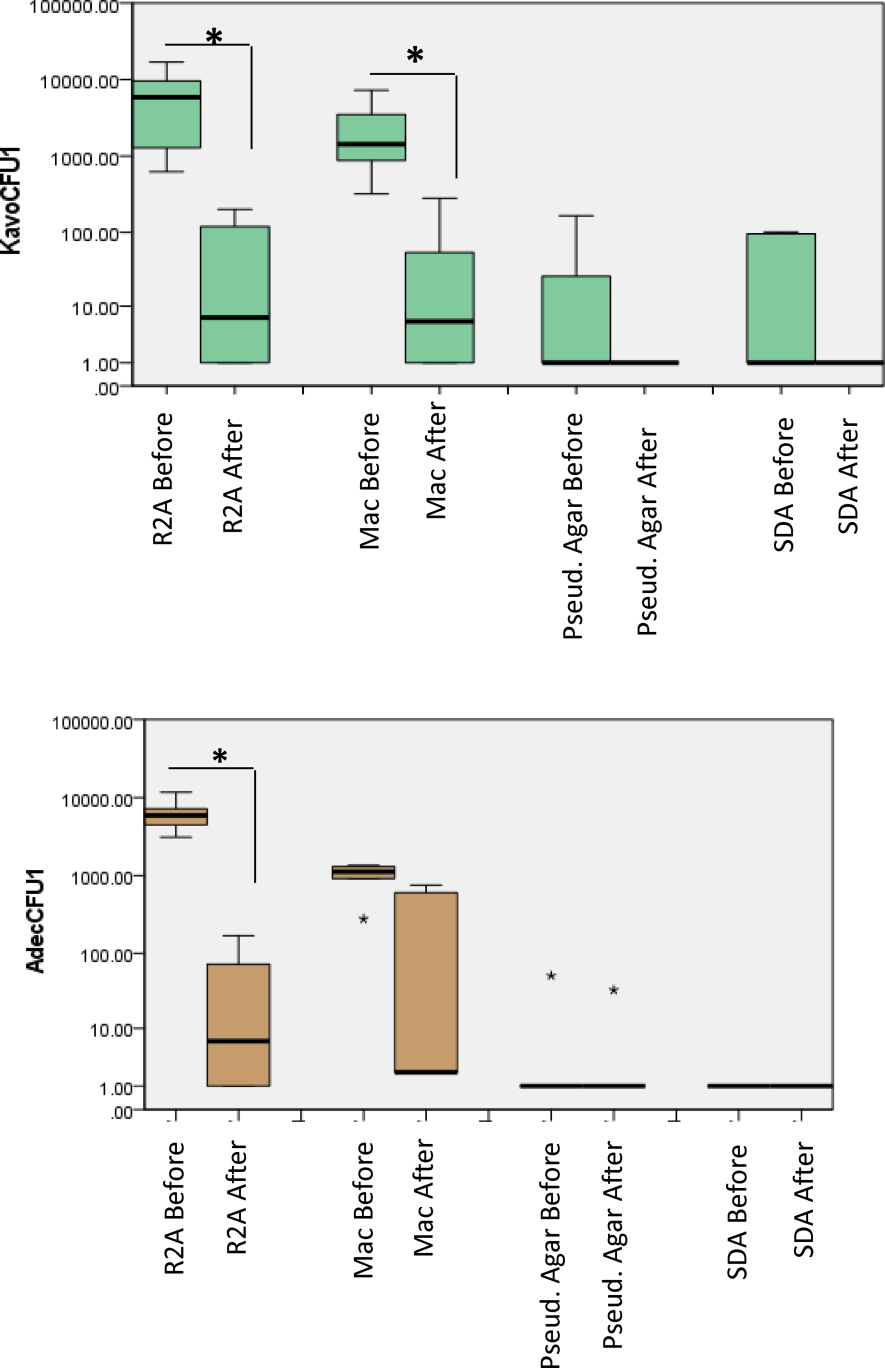
Total viable counts from water samples collected from dental unit hand pieces. Samples were collected before and after treatment, 10-fold serially diluted up to 10^−5^ and plated on different culture media. The plates were then incubated in different atmospheric conditions for different incubation times as described in “methods”. *P* < 0.05 Mann Whitney U.

The mean (SD) CFU counts were the highest from the R2A medium in both A-dec and KaVo units. The CFU counts from A-dec were, 6.38 × 10^4^ (5.7 × 10^2^), which was significantly (*P* < 0.05) reduced to 4.41 × 10^2^ (2.2 × 10^2^) after the disinfection treatment procedure (Figure 2). Similarly, from KaVo, the mean (SD) CFU counts decreased from 8.8 × 10^3^ (1.3 × 10^3^) to 2.5 × 10^2^ (2.2 × 10^1^).

From the MacConkey agar, the mean (SD) CFU counts decreased from 1.1 × 10^3^ (1.14 × 10^2^) to 2.2 × 10^2^ (2 × 10^2^) for A-dec and from 5.18 × 10^3^ (1.0 × 10^2^) to 2.7 × 10^2^ (2.2 × 10^1^) in the case of KaVo. Bacterial growth was observed on Pseudomonas agar from only one of the dental units from each of A-dec [5 × 10^2^ (2.8 × 10^1^)], which were reduced to 3.2 × 10^1^ (2.54 × 10^1^). For KaVo, the mean (SD) CFU counts from Pseudomonas agar were [1 × 10^2^ (1.4 × 10^1^)] which decreased to zero counts after treatment.

### Waterline contamination of storage water bottles

**Figure 3:**
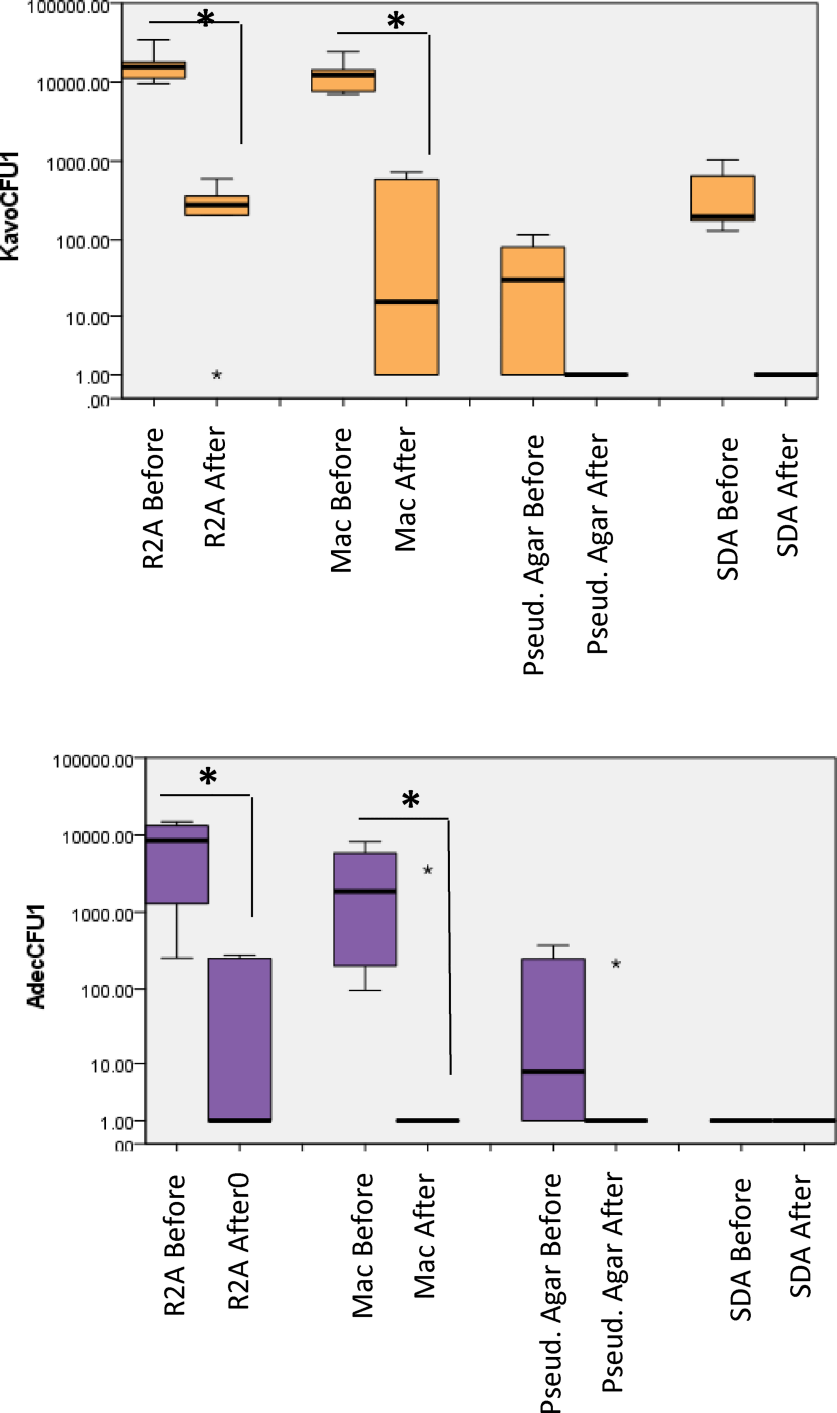
Total viable counts from water samples collected from dental unit water storage bottles. Samples were collected before and after treatment, 10-fold serially diluted up to 10^−5^ and plated on different culture media. The plates were then incubated in different atmospheric conditions for different incubation times as described in “methods”. *P* < 0.05 Mann Whitney U.

Similar to handpieces, highest CFU counts were observed from R2A medium in the case of water samples from the storage bottles at each unit (Figure 3). For A-dec, the mean (SD) counts decreased from 7.78 × 10^3^ (8.2 × 10^2^) 8.6 × 10^1^ (3.4 × 10^1^). MacConkey agar: the CFU counts 3.1 × 10^3^ (3.8 × 10^2^) decreased to 5.8 × 10^2^ (5.3 × 10^2^) for A-dec. For KaVo, the counts were about 1 log higher, i.e., 1.3 × 10^4^ (1.9 × 10^3^) which were decreased to 2.6 × 10^1^ (7.42 × 10^0^) (*P* < 0.05). Most of the water storage bottles (4–5) from A-dec and KaVo showed colonies on Pseudomonas agar. For A-dec, the mean (SD) CFUs on Pseudomonas agar were 1.8 × 10^2^ (2.21 × 10^1^) which decreased to 3.5 × 10^1^ (3.6 × 10^1^) while the mean counts of 7.75 × 10^1^ (4.6 × 10^1^) decreased to zero counts in all units.

### Biofilm growth on the tubings

**Figure 4:**
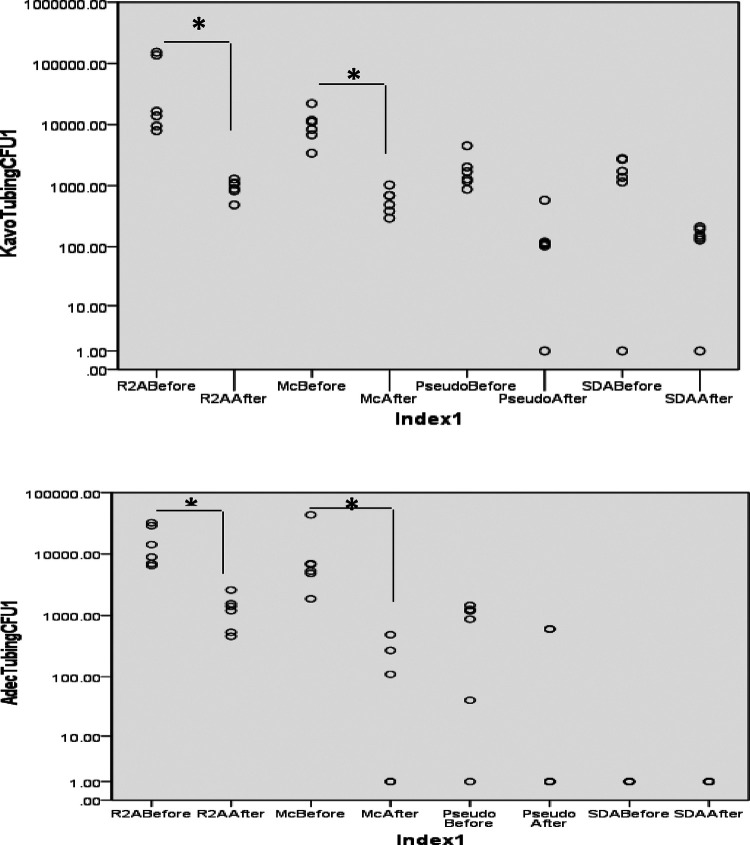
Bacterial counts from the biofilm harvested from DUWL tubing before and after treatment. Biofilm was scraped off the tubing surface using sterile dental probe and suspended in sterile PBS. Serial 10-fold dilutions (up to 10^−5^) were prepared and plated on different culture media. *P* < 0.05 Mann Whitney U.

The bacterial load in the biofilms growing on the inner surface of the dental waterline tubings was also determined by microbiological culture (Figure 4). The mean (SD) CFU counts on were 1.64 × 10^4^ (1.1 × 10^4^) which decreased to 1.2 × 10^3^ (7.8 × 10^2^) on R2A medium, MacConkey agar: 1.15 × 10^4^ (1.5 × 10^4^) decreased to 1.4 × 10^2^ (1.9 × 10^2^); Pseudomonas agar: 8 × 10^2^ (6.3 × 10^2^) decreased to 2 × 10^2^ (3 × 10^2^). No growth was observed on SDA medium.

From the KaVo units, microbial growth was observed on all culture media. The mean (SD) CFU counts were 5.6 × 10^4^ (6.85 × 10^4^) and showed a significant decrease to 9.4 × 10^2^ (2.78 × 10^2^) (*P* < 0.05); MacConkey agar: 1.06 × 10^4^ (6.3 × 10^3^) decreased to 5.95 × 10^2^ (2.6 × 10^2^); Pseudomonas agar: 1.9 × 10^3^ (1.3 × 10^3^) decreased to 1.7 × 10^2^ (2 × 10^2^). Colonies typical of Candida species were observed on SDA medium, 1.6 × 10^3^ (1 × 10^3^) decreased to 1.3 × 10^2^ (7.4 × 10^1^).

### Identification of the predominant bacterial species from dental unit waterlines by 16s rRNA gene sequencing

16S rRNA gene sequencing was used to identify the most-often occurring colony-types across all growth media (Table 1). Majority of the identified species were Gram-positive while only 5 were Gram-negative. Of the Gram-positives, important species from the *Staphylococcus* genera, *Staphylococcus pasteuri, Staphylococcus warneri, Staphylococcus hominis, Staphylococcus hominis* and *Staphylococcus captis* were identified. Some of the other Gram-positive species were *Micrococcus luteus, Corynebacterium aurimucosum, Corynebacterium singular, Corynebacterium amycolaturm,* and *Bacillus subtilis*. Interestingly, the oral species *Streptococcus salivarius* was also present in the identified species. *Acinetobacter indicus, Paenibacillus lactis, Roseomonas mucosa Roseomonas aerofrigidensis* were the Gram-negative species identified from the waterline samples.

**Table 1:** Predominant microbial species in the DUWL identified by 16S rRNA gene sequencing. GenBank submission ID: 2641990.

S. No.	Species
1	*Acinetobacter indicus*
2	*Bacillus glycinifermentans*
3	*Bacillus subtilis*
4	*Bacillus tequilensis*
5	*Corynebacterium amycolatum*
6	*Corynebacterium aurimucosum*
7	*Corynebacterium singulare*
8	*Exiguobacterium mexicanum*
9	*Kocuria rhizophila*
10	*Micrococcus luteus*
11	*Paenibacillus lactis*
12	*Paracraurococcus* sp*.*
13	*Roseomonas aerofrigidensis*
14	*Roseomonas mucosa*
15	*Staphylococcus aureus*
16	*Staphylococcus borealis*
17	*Staphylococcus capitis*
18	*Staphylococcus hemolyticus*
19	*Staphylococcus hominis*
20	*Staphylococcus pasteuri*
21	*Staphylococcus warnerii*
22	*Streptococcus salivarius*
23	*Terribacillus halophilus*

## Discussion

The microbiological assessment of the samples before and after the treatment intervention of the dental unit waterlines showed a significant reduction in the microbial colony counts following the treatment procedure. Importantly, 16S rRNA gene-based identification of the major colony-types revealed that the waterlines were home to numerous potentially hazardous bacterial species.

We used four different types of microbiological culture media, R2A for cultivation and enumeration of environmental bacteria from potable water in laboratory settings, MacConkey medium for selective growth of Gram-negative bacteria, Pseudomonas agar with CFC supplement was intended for selective isolation of *Pseudomonas* species (15). SDA medium with chloramphenicol was used for *Candida* species (16). An important limitation of this study was that we did not attempt to culture *Legionella* spp., an important human pathogen that frequently occurs in DUWL (17, 18). From all types of samples, water from handpieces and the storage bottles, and from the tubings, R2A medium followed by MacConkey medium, demonstrated highest number of CFUs. Following treatment for 15 days, the CFU counts were significantly reduced to about 10^2^–10^3^ CFUs. Bacterial colonies that grew on Pseudomonas agar did not belong to the *Pseudomonas* species as per f16S rRNA gene sequencing. It is possible that the Pseudomonas agar we used was not selective and some other species grew on it. Colonies from SDA medium appeared to be Candida species, which were further characterized by Gram-staining.

Importantly, the luminal surface of the waterline tubings harbored dense biofilm as seen in the scanning electron microscopy images. Upon culturing the scraped biofilm from the tubing, a heavy bacterial growth was observed on R2A agar and MacConkey medium, while lesser growth was seen on the other culture media. It is believed that once macromolecules in the water adhere to the luminal surface of the tubings and a conditioning film is formed, microorganisms may attach to such a surface. Subsequently, the attached bacteria divide and the planktonic cells in water begin to attach to the surface, leading to the initiation of biofilm formation (19). As the biofilms mature, they break-open resulting in the release of free bacterial cells inside the biofilm into the flowing water (20). In the absence of an effective disinfection procedure, the bacterial cells released from mature biofilms find new surfaces to adhere and begin new biofilm colonies. In our study, the disinfection treatment resulted in significant reduction in the microbial counts on all growth media, attesting the efficacy of the treatment method we used.

In the dental clinics, a variety of disinfectants have been used for DUWL maintenance. Most are based on sodium hypochlorite (NaOCl), hydrogen peroxide (H_2_O_2_) – with or without silver ions – and the TAED (tetra-acetylethylenediamine) formulation of peracetic acid (21). All of them can be used, either periodically (highly concentrated shock treatment) or continuously (less concentrated treatment). Neither approach is devoid of shortcomings. While periodic treatments result in recolonization within 2–3 weeks after the treatment is stopped, continuous uninterrupted treatment may expose the patients and the staff to unintended biocide exposure (22). More importantly, prolonged use of disinfectants may lead to the emergence of tolerant or resistant species within the DUWL biofilms (23). In our study, even though we used a chemical disinfectant on an intermittent basis, we implemented surrogate practices that may help prevent recolonization of the waterlines by microorganisms. At the end of the day, all the water that had accumulated in the waterlines was drained. At the start of each day, the hand piece, three-in-one syringe, and other instruments connected to the dental unit waterline were flushed for 5 min to eradicate any bacteria still present in the tubing. Similar study several years ago conducted at the Government Polyclinics was shown to be highly effective in controlling the microbial contamination in DUWL (13).

16S rRNA gene-based identification of the most commonly occurring colony-types across all media revealed a number of bacterial species that may be potentially pathogenic. Strains from *Staphylococcus* species such as *S. aureus*, *S. warneri*, *S. hominis*, and *S. haemolyticus* were reported to be methicillin-resistant (24). *Roseomonas mucosa* (25), *Paenibacillus* spp (26), *Kocuria rhizophila* (27), *Acinetobacter indicus* (28), and *Bacillus subtilis* (29). Remarkably, several of the identified species are recognized as pathogens in immunocompromised patients. Further, several species identified here have also been reported to be occurring in the dental unit waterlines in previous studies (30). More recent literature on comprehensive microbiota analyses of the DUWL have shed more light on the microbial diversity of the DUWL biofilms.

## Conclusion

In conclusion, our study demonstrated that periodic disinfection of the dental unit waterlines by following manufacturer instructions alone may not be sufficient in keeping the microbial counts low. A comprehensive treatment of the waterlines was found to be effective in reducing the microbial counts to the levels permissible by the ADA: Flushing the waterline tubings with disinfectant, discarding the left-over water in the storage bottles, draining the tubings daily, flushing the handpiece and other instruments attached to the dental unit daily at the beginning. This may minimize the risk of infections for both the dental unit staff and the patients.

## Data Availability

The datasets presented in this study can be found in online repositories. The names of the repository/repositories and accession number(s) can be found below: https://www.ncbi.nlm.nih.gov/genbank/, 2641990.
